# The effect of disease severity markers on quality of life in autosomal dominant polycystic kidney disease: a systematic review, meta-analysis and meta-regression

**DOI:** 10.1186/s12882-017-0578-6

**Published:** 2017-05-25

**Authors:** Myrte K. Neijenhuis, Wietske Kievit, Ronald D. Perrone, Jeff A. Sloan, Patricia Erwin, Mohammad Hassan Murad, Tom J. G. Gevers, Marie C. Hogan, Joost P. H. Drenth

**Affiliations:** 1 Department of Gastroenterology and Hepatology, Radboud University Medical Center, P.O. Box 9101, code 455, 6500 HB Nijmegen, the Netherlands; 2 Radboud Institute for Health Science, Radboud University Medical Center, Nijmegen, the Netherlands; 3 Department of Medicine, Division of Nephrology, Tufts Medical Center, Boston, MA USA; 4 Department of Health Sciences Research, Mayo Clinic, Rochester, MN USA; 5 Mayo Clinic Libraries, Mayo Clinic, Rochester, MN USA; 6 Robert D. and Patricia E. Kern Center for the Science of Health Care Delivery, Mayo Clinic, Rochester, MN USA; 7 Division of Nephrology and Hypertension, Department of Internal Medicine, Mayo Clinic, Rochester, MN USA

**Keywords:** Autosomal dominant polycystic kidney disease, Quality of life, Renal function, Kidney volume, Liver volume

## Abstract

**Background:**

Little is known about determinants of quality of life (QoL) in autosomal dominant polycystic kidney disease (ADPKD). Recent studies suggest that QoL in ADPKD is determined by more factors than mere renal function. We investigated the effect of ADPKD on QoL and evaluated how Qol is affected by disease severity markers renal function, kidney volume and liver volume.

**Methods:**

We performed a systematic review, meta-analysis and meta-regression analyses of cohort studies and randomized controlled trials investigating patient-reported QoL in adult patients with ADPKD not yet on dialysis. EMBASE, MEDLINE, and Web of Science were searched to August 2015 without language restrictions. Two investigators independently reviewed title, abstracts and full text of potentially relevant citations to determine eligibility. We compared pooled QoL summary scores of ADPKD patients using a random-effects meta-analytic model. These scores were compared with mean and age-corrected reference scores of the general population. In a meta-regression analysis, we investigated the univariate effect of renal function, kidney volume and liver volume on QoL.

**Results:**

We included nine studies in meta-analysis including 1623 patients who completed the SF-36 questionnaire. Pooled physical (PCS) and mental component scores (MCS) of the SF-36 of individuals with ADPKD were lower than those of the reference population (45.7 vs. 50.0 and 47.8 vs. 50.0 points, both *P* < 0.001). QoL of ADPKD patients remained lower after comparison with age-corrected reference values (age 35–44 year; PCS 52.2, MCS 49.9 points, both *P* < 0.05). Larger liver volume negatively impacted PCS (*P* < 0.001) and MCS (*P* = 0.001), whereas there was no association with renal function (PCS *P* = 0.1, MCS *P* = 0.9) and kidney volume (PCS *P* = 0.5, MCS *P* = 0. 5). Total liver and kidney volume had no impact on PCS (*P* = 0.1), but did have impact on MCS (*P* = 0.02).

**Conclusions:**

QoL reported by non-dialysis patients with ADPKD is impaired compared to the general population. Large liver volume was the most important factor that diminishes QoL.

PROSPERO International Registry number CRD42015026428.

**Electronic supplementary material:**

The online version of this article (doi:10.1186/s12882-017-0578-6) contains supplementary material, which is available to authorized users.

## Background

A growing body of evidence on QoL in chronic kidney disease (CKD) suggests that quality of life (QoL) is not determined merely by renal function [1–3]. The presence of anemia and cardiovascular disease can also have substantial negative impact on QoL [1–3]. In autosomal dominant polycystic kidney disease (ADPKD), anemia [4, 5], and cardiovascular diseases [6] are less frequent compared to other kidney diseases. This suggests that other disease-specific factors may contribute to QoL impairment in ADPKD [7].

ADPKD is defined by progressive renal cyst development, leading to enlarged kidneys, kidney failure and eventually end stage kidney disease [8]. Kidney manifestations of ADPKD include acute and chronic pain, hematuria, nephrolithiasis, and cyst infection [9]. This disease is associated with a range of extrarenal manifestations, including liver cysts which occur in the majority of patients, mitral valve abnormalities and intracranial aneurysms [10]. Clinical symptoms in ADPKD seems to be a function of kidney and liver size, and are not primarily related to kidney function decline [11].

Large kidney and liver volumes compress adjacent organs and structures leading to symptoms that may impair QoL such as fullness, early satiety and pain [12, 13]. Further, it has been demonstrated that kidney enlargement occurs well before renal function decline and severe polycystic liver disease may also occur in early stage ADPKD [8, 12, 14]. This indicates that physical manifestations of ADPKD which may adversely affect QoL are present before renal function deficits are detected [8, 13].

How disease severity markers, such as renal function, kidney volume and liver volume, affect QoL in ADPKD is uncertain. Several studies showed at best very weak correlations among these variables [12–16]. Most studies included a selected population of patients with ADPKD based on either chronic kidney disease (CKD) stage or organ volumes which limits drawing robust conclusions on the relation between disease severity markers and QoL across the clinical spectrum of ADPKD. Understanding factors that impact QoL of individuals with ADPKD can guide treatment decisions and improve holistic patient-centered care beyond simply monitoring renal function [7].

This systematic review and meta-analysis investigates the effect of ADPKD on QoL. As secondary outcome, we assessed the effect of the disease severity markers renal function, kidney volume and liver volume on QoL in ADPKD.

## Methods

This systematic review was conducted according to a research protocol registered in the PROSPERO international prospective register of systematic reviews (registration number CRD42015026428). We reported this systematic review in accordance with the Preferred Reporting Items for Systematic Reviews and Meta-Analyses (PRISMA) guidelines [17].

### Eligibility criteria

We included studies that met the following inclusion criteria: (1) cohort studies and randomized controlled trials (RCTs); (2) adult patients >18 years, with a diagnosis of ADPKD [18] and; (3) use of a patient-reported outcome to reflect QoL. We excluded studies that (1) used a patient-reported outcome without summary score of individual questions, (2) studies that investigated QoL with a one-item visual analogue scale (VAS) only as it often provides insufficient QoL information [19], (3) longitudinal intervention studies that provided no baseline QoL scores and, (4) studies that did not report original data.

### Search strategy

A medical librarian (P.E.) developed and executed a systematic search combining the search terms and Medical Subject Headings for ‘ADPKD’ and ‘renal function’ or ‘kidney volume’ or ‘liver volume’ and ‘quality of life’ in the electronic databases of EMBASE, MEDLINE, and Web of Science. Letters to the editor, editorials and case reports were excluded. Additional file 1: Table S1 provides an example of one full electronic search. We searched for conference abstracts in abstract books of the American Society of Nephrology (ASN), World Congress of Nephrology (WCN) and the European Renal Association – European Transplant and Dialysis Association (ERA-EDTA) published between August 2012 and August 2015 and unpublished studies in the database of clinicaltrials.gov. Reference lists of included articles and relevant reviews were screened for additional leads. Two investigators (M.N. and M.H.) independently reviewed title and abstracts to determine eligibility. Disagreements between M.N and M.H. were included for full text review. Subsequently, both investigators screened full text of eligible studies and disagreement between M.N and M.H. was resolved by discussion with a third author (T.G.). Corresponding authors of original articles were contacted for additional information if needed. Cohen’s kappa was calculated as measure of agreement on study selection. A value between 0.40–0.59 was considered as fair agreement, 0.60–0.74 as good agreement and 0.75 or higher as excellent agreement [20].

### Quality assessment

Since there is no standard tool available to assess the risk of bias for uncontrolled studies, we used items derived from the Newcastle-Ottawa Scale to assess risk of bias on study and outcome level [21]. We scored the categories (1) study population selection; (2) completeness of reported results; (3) used patient-reported outcome instrument; (4) recall period and; (5) response rate as low or high risk of bias followed by an overall conclusion of the risk of bias (low, moderate or high risk of bias) as judged by two independent reviewers.

### Outcome

Primary outcome was summary QoL score measured with a patient-reported outcome instrument at baseline. We included studies in meta-analysis and meta regression that used a patient-reported outcome measure that was used in 3 or more studies [22]. Scores of individuals with ADPKD were compared with reference values if available.

We expected that the SF-36 was used in most studies, as it is the most frequently used patient-reported outcome worldwide and is often used in kidney studies [23, 24]. The SF-36 is a generic QoL measure that composes of eight domains (physical functioning, role-physical, bodily pain, general health, social functioning, vitality, role-emotional, and mental health) that can be summarized in two composite scales; the physical component scale (PCS) and mental component scale (MCS) [25]. These summary scores can reduce type I errors by avoiding multiple testing and can distinguish better between different health state levels than the eight separate domains [26]. A higher PCS or MCS score indicate a better QoL. Population-based reference values for both component scales are set to 50 points with a standard deviation of 10 points [27]. We calculated component scores of the SF-36 using US norm values, as previous studies showed a similar impact of chronic diseases on QoL across different countries [28].

### Data extraction

M.N. extracted data of the included articles using a standardized data collection form to record (1) study characteristics: first author, year of publication, country of origin, study design and number of participants; (2) patient characteristics: age, gender, chronic kidney disease (CKD) stage as defined by KDIGO [29], mean renal function defined as (estimated) GFR in ml/min/1.73m^2^, median kidney and liver volume measured with volumetric software or ellipsoid formula (kidney volume only); (3) QoL data: baseline summary scores of patient-reported outcome instruments. For studies that presented only serum creatinine as measure of renal function, we calculated the eGFR with the chronic kidney disease epidemiology collaboration (CKD-EPI) formula and Modification of Diet in Renal Disease (MDRD) formula [30]. Total liver and kidney volume was calculated by summing liver and kidney volumes. In RCTs, baseline QoL scores of both placebo and intervention groups of RCTs were pooled. QoL data of subgroups stratified by renal function was handled separately. Summary scores were recalculated when the original authors used a scoring algorithm which was different from the official published scoring manual. W.K. reviewed the complete dataset for completeness and accuracy.

### Data analysis

To calculate the effect of ADPKD on QoL, we performed a pre-specified analysis using a random-effects meta-analytic model of DerSimonian and Laird [31]. The random effect model is used if large heterogeneity is expected and adjusts for differences in study size. Heterogeneity was assessed using I^2^ statistics and a cut-off value of I^2^ > 50% was considered as substantial heterogeneity [32]. When population-based reference values of included patient-reported outcomes were available, we compared whether the pooled mean scores of ADPKD patients were significantly different compared to the general population with an independent t-test. When the pooled mean of ADPKD patients was significantly lower than the general population, we compared the pooled means with age-corrected values to assess whether this difference remained significant. Additional sensitivity analyses including high quality studies only were performed to assess the influence of study quality.

To explore the effect of disease severity markers on QoL, we performed a pre-specified univariate meta-regression analysis in which the dependent variable was QoL score (PCS or MCS) and the independent variables were renal function, kidney volume, liver volume and total liver and kidney volume. As additional exploratory analysis, we performed the primary meta-regression excluding studies with severe liver involvement (defined as median liver volume > 3000 mL, based on a previous published classification [14]). Kidney and liver volumes have a skewed distribution and were logarithmically transformed. If a study provided no standard deviation (SD), standard error (SE) or interquartile range (IQR) of the summary QoL score, we imputed SD from the mean SD of other included studies. As a sensitivity analysis, we compared the results of the meta-regression of eGFR calculated with the CKD-EPI with eGFR calculated with the MDRD formula. Analyses were performed using the statistical program OpenMetaAnalyst [33]. For all analyses, *P* < 0.05 was considered statistically significant.

## Results

**Fig. 1:**
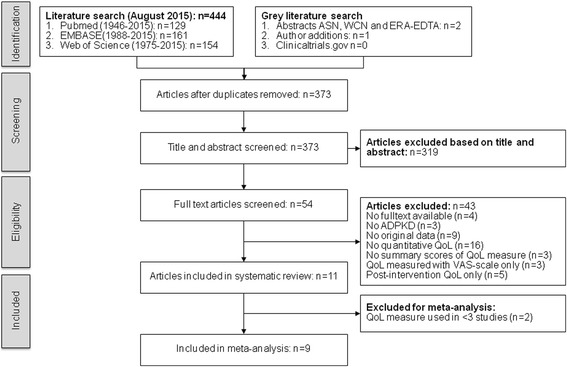
PRISMA Flow-diagram of study inclusion

From a total of 373 unique articles identified by our systematic search, 11 studies matched our inclusion criteria [13, 15, 16, 34–41]. Figure 1 shows the PRISMA flow diagram with specified reasons for exclusion. There was excellent agreement between the two reviewers on study selection (Cohen’s Kappa 0.89). We found only two studies that investigated QoL in patients with ADPKD on dialysis (*n* = 108 and *n* = 5 respectively), which prevented a reliable meta-analysis in this subgroup [15, 34]. We contacted eight authors for additional information that was not published in the original articles and received relevant data from six of them [15, 34–36, 38, 39]. Four studies included a mixed population of patients with isolated polycystic liver disease (ADPLD) and polycystic liver disease as extrarenal manifestation of ADPKD [35, 36, 39, 40]. For all these studies, we obtained separate ADPKD group data by contacting the authors.

Eight studies used the SF-36 as patient-reported outcome and one study used the KDQOL-SF1.3, a combined questionnaire of the SF-36 and 43 kidney disease-specific questions. The gastrointestinal symptom scale was administrated together with the SF-36 or EQ-5D in three studies [36, 40, 41]. As there is no validated total score of the gastrointestinal symptoms scale and the EQ-5D was used in only two studies, this resulted in exclusion of two studies for meta-analysis [40, 41]. Other patient-reported outcomes administrated together with the SF-36 were used in less than three studies and were also excluded from further analyses (see Table 1). After applying these eligibility criteria, only studies that used SF-36 data were eligible for inclusion.

**Table 1:** Characteristics of included studies

First author, year	Country of origin	Study design	(Sub)group	Patients (n), completed PRO%	Age (mean ± SD)	Gender (% male)	CKD stage	(e)GFR ml/min/1,73m^2^(mean ± SD)	Kidney volume (mL), (mean ± SD or median (IQR))	Liver volume (mL) (mean ± SD or median (IQR))	Patient-reported outcome measure
Barros et al., 2011 [34]	Brazil	Cross-sectional	Non-dialysis	33 (100)	37 ± 12	24	1–4	69 ± NR^a^	NR	NR	State Trait Anxiety Inventory, Beck Depression scale, SF-36
Hogan et al., 2010 [35]	USA	Randomized controlled trial	All patients	34 (100)	50 ± 9	24	1–4	61 ± 22	852 (486–1430)	4969 (3258–8404)	SF-36
Keimpema et al., 2009 [36]	Netherlands	Randomized controlled trial	All patients	32 (97)	51 ± 9	13	1–4	64 ± 25	872 (502–1551)	4906 (3380–6416)	SF-36, Gastrointestinal symptom scale^b^
Lee et al., 2003 [37]	USA	Longitudinal observational study	All patients	29 (28)	46 ± 14	24	1–4	NR	2276 (NR)	NR	SF-36
Miskulin et al., 2014 [13]	USA	Randomized controlled trial	All patients	1043 (99)	42 ± NR	50	1–4	68 ± NR	1215 ± NR^c^	1958 ± NR^c^	SF-36, Modified version of the Wisconsin Brief pain survey
			eGFR 20–44	213	49 ± 8	50	3–4	37 ± 5	NR	NR	
			eGFR 45–60	221	47 ± 8	51	3	52 ± 5	1821 ± 1041	2225 ± 790	
			eGFR ≥ 60	609	37 ± 9	50	1–2	85 ± 18	1198 ± 711	1950 ± 800	
Rizk et al., 2009 [16]	USA	Longitudinal observational study	All patients	152 (100)	44 ± 10	40	1–4	65 ± 33	937 ± 883	NR	SF-36
Simms et al., 2015 [38]	United Kingdom	Cross-sectional	All patients	139 (100)	49 ± 16	48	1–4	59 ± 33	NR	NR	KDQOL-SF1.3, Patient health questionnaire (PHQ-9), multidimensional scale of perceived social support (MSPSS), Sheffield ADPKD psychosocial risk instrument (Sheffield ADPKD PSRI) SF-36 and self-made symptom scale
			eGFR ˂30	36	66 ± 13	47	4	18 ± 5	NR	NR	
			eGFR 30–60	38	56 ± 11	47	3	45 ± 8	NR	NR	
			eGFR > 60	65	45 ± 14	35	1–2	89 ± 19	NR	NR	
Suwabe et al., 2013 [15]	Japan	Longitudinal observational study	Non-dialysis	111 (100)	52 ± 12	40		30 ± NR^a^	3308 ± 2566	3841 ± 3551	
Temmerman et al., 2014 [39]	Belgium	Longitudinal observational study		50 (100)	51 ± 9	10	1–4	58 ± 24	1126 (586–1806)	4918 (3942–6360)	SF-36, Polycystic liver diseasecomplaint-specific-assessment (POLCA)

*CKD* chronic kidney disease stage, *NA* not applicable, *NR* not reported, *PRO* patient reported outcome, *SD* standard deviation ^a^Calculated based on serum creatinine with CKD-EPI formula ^b^No validated total score available ^c^Kidney and liver volumes only measured in patients with eGFR >45 ml/min

### Characteristics and quality of included studies

Table 1 describes the characteristics of the nine studies included in meta-analysis [13, 15, 16, 34–39]. The majority were longitudinal interventional studies (*n* = 7), including three randomized controlled trials. The nine studies included 1623 non-dialysis patients. At least 753 patients had CKD stage 1–2 and 478 CKD 3–4. There was insufficient individual patient data on the renal function of 363 patients to distinguish between CKD stage 1–4. One study (*n* = 29) did not provide renal function of the included patients [37], resulting in 1594 available patients to assess the impact of renal function on QoL. Seven studies reported kidney volumes (*n* = 1238) of the included patients [13, 15, 16, 35–37, 39]. Five studies reported liver volume (*n* = 1057), [13, 15, 35, 36, 39] including four studies with severe polycystic liver disease patients (median liver volume > 3000 mL) [15, 35, 36, 39]. The SD of the PCS and MCS was imputed in four studies. Patients were on average 44 years and 45% was male. Pooled mean (e)GFR was 58 mL/min/1,73m^2^ (95% CI 47 to 69), kidney and liver volumes were respectively 1465 mL (95% CI 1146 to 1784) and 3599 mL (95% CI 3010 to 4187). This indicates enlargements of approximately 4 and 2.5 times compared to normal kidneys (300 mL for females and 400 mL for males) and livers (1400 mL for females, 1700 mL for males), respectively.

A detailed quality assessment of the studies is presented in Additional file 1: Table S2. There was a low risk of bias in the majority of the studies (*n* = 8) and one study was rated as having a high risk of bias (insufficient information on study population selection and very low response rate).

### QoL in ADPKD

**Fig. 2:**
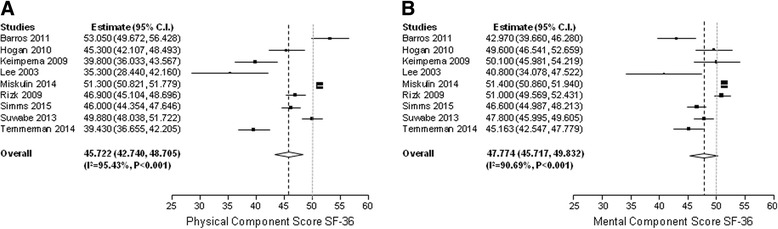
Pooled physical (**a**) and mental (**b**) component score of the SF-36 of individuals with ADPKD. ADPKD patients (*black line*) scored lower than the general population (*grey line*)

Figure 2 shows the results of the pooled SF-36 scores of the PCS (A) and MCS (B). The mean PCS of individuals with ADPKD was 45.7 points (95% CI 42.7 to 48.7), although there was significant heterogeneity (I^2^ 95.4%, *P* < 0.001). This was significantly different from the mean score of the general population (PCS 50 points 95% CI 49.6 to 50.4, *P* < 0.001). On the MCS, patients scored 47.8 points (95% CI 45.7–49.8), with again large heterogeneity (I^2^ 90.7%, *P* < 0.001). Also this score was lower compared to the general population (PCS 50 points, 95% CI 49.6 to 50.4, *P* < 0.05). Compared with age-corrected reference values (age 35–44 year; (PCS 52.2, 95% CI 51.5 to 52.8 and MCS 49.9 points, 95% CI 49.1 to 50.7, both *P* < 0.001), QoL of ADPKD patients remained significantly lower. Sensitivity analysis including high quality studies only showed no differences in pooled scores compared to the analyses including all studies.

### The relationship between markers of disease severity and QoL in APDKD patients

**Fig. 3:**
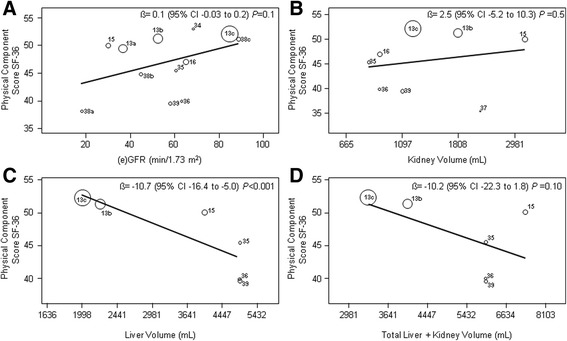
Meta-regression analysis of the physical component score of the SF-36 with the factors (**a**) (e)GFR (ml/min/1.73m^2^), **b** Kidney volume, **c** Liver volume and **d** Total liver and kidney volume. Volumes are presented on a logarithmic scale. Barros et al., [34]; Hogan et al., [35]; Keimpema et al., [36]; Lee et al., [37]; Miskulin et al., [13] subgroup eGFR 20–44*; Miskulin et al., [13] subgroup eGFR 45–60; Miskulin et al., [13] subgroup eGFR ≥60; Rizk et al., [16]; Simms et al., [38] subgroup eGFR <30; Simms et al., [38] subgroup eGFR 30–60; Simms et al., [38] subgroup eGFR >60; Suwabe et al., [15]; Temmerman et al., [39]. *No kidney and liver imaging in this subgroup, not included in figures **b**-**d**

The relationship of the disease severity markers renal function, kidney volume, liver volume and total liver and kidney volume on PCS is shown in Fig. 3. Larger liver volume negatively impacted PCS (ß = −10.7, 95% CI -16.4 to −5.0, *P* < 0.001). We observed no significant effect of renal function (ß = 0.1, 95% CI -0.03 to 0.2, *P* = 0.1), kidney volume (ß = 2.5, 95% CI -5.2 to 10.3, *P* = 0.5) and total liver and kidney volume (ß = −10.2, 95% CI -22.3 to 1.8, *P* = 0.10). On MCS, larger liver volume (ß = −4.7, 95% CI -7.5 to −1.8, *P* = 0.001) and total liver and kidney volume (ß = −5.6, 95% CI -10.0 to −1.1, *P* = 0.02) had significant impact, while renal function (ß = −0.005, 95% CI -0.08 to 0.07, *P* = 0.9) and kidney volume (ß = −1.5, 95% CI -5.6 to 2.5, *P* = 0. 5) did not (Additional file 2: Figure S1). Sensitivity analyses showed similar results using the MDRD formula to calculate eGFR or when low quality studies were excluded.

**Fig. 4:**
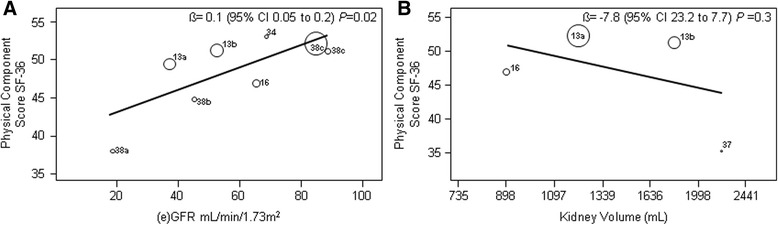
Meta-regression analysis of the physical component score of the SF-36 with the factors (**a**) (e)GFR (ml/min/1.73m^2^) and **b** Kidney volume after exclusion of studies with severe liver involvement. Volumes are presented on a logarithmic scale. Barros et al., [34]; Lee et al., [37]; Miskulin et al., [13] subgroup eGFR 20–44*; Miskulin et al., [13] subgroup eGFR 45–60; Miskulin et al., [13] subgroup eGFR ≥60; Rizk et al., [16]; Simms et al., [38] subgroup eGFR <30; Simms et al., [38] subgroup eGFR 30–60; Simms et al., [38] subgroup eGFR >60. *No kidney and liver imaging in this subgroup, not included in figure **b**

In studies with mild to moderate liver involvement (median liver volume ≤ 3000 mL), renal function did have a negative impact on PCS (ß = 0.1, 95% CI 0.05 to 0.2, *P* = 0.02), but there was no effect of kidney volume (ß = −7.8, 95% CI 23.2 to 7.7, *P* = 0.3; Fig. 4). On the MCS, neither renal function (ß = −0.02, 95% CI -0.11 to 0.08, *P* = 0.8) nor kidney volume (ß = −2.9, 95% CI -10.2 to 4.3, *P* = 0.4) had impact (Additional file 3: Figure S2).

## Discussion

We show that ADPKD negatively impacts QoL, particularly through the effect of the disease on parameters of the physical domain. Individuals with ADPKD had lower PCS and MCS compared to population-based reference values. We found large heterogeneity across QoL of individuals with ADPKD. In the primary meta-regression performed to explain this heterogeneity, larger liver volume, but not renal function and kidney volume, correlated with lower physical and mental components of health status in ADPKD. However, after exclusion of studies of patients with large liver volumes, lower renal function was also correlated with lower PCS.

Large liver volume had a negative effect on QoL in ADPKD, indicating that liver volume is an important parameter that should be accounted for when investigating QoL in this population. However, the strong female predominance in severe polycystic liver disease studies (74 vs. 53%, *P* < 0.001) and inclusion of slightly older patients (mean age 51 ± 10 vs. 43 ± 12 year, *P* = 0.24) in studies with mild to moderate liver involvement can also contribute to QoL impairment in these patients [27]. Comparing general reference values of younger males (35–44 years) with older females (45–54 years), resulted in a mean difference of 4.5 and 0.2 points for PCS and MCS, respectively. The loss of QoL in the liver volume meta-regression analysis was larger than these differences (ß = −10.7 points per logarithm for PCS and ß = −4.7 for MCS), suggesting an independent additional effect of liver volume on QoL.

Our exploratory analysis suggests that QoL declines during progression towards CKD stage 5 in patients without severe polycystic liver disease. This indicates that renal function is also an important factor of QoL in ADPKD, but this effect was negated by the strong impact of liver volume in the total group. An independent patient data analysis with correction for liver volume is necessary to draw definitive conclusions about the role of renal function and kidney volume on QoL.

A previous study showed that patients with larger kidney volume reported more pain that impacted their daily life compared to patients with smaller kidneys [13]. We did not find a significant impact of kidney volume on QoL, possibly because only a limited number of studies in our meta-analysis included patients with large kidney volumes. A large observational study including 450 ADPKD patients of CKD stages 1–4 (DIPAK) is currently being conducted in the Netherlands. This study may reveal whether large kidney volume contributes to a decrease in QoL.

A strength of this study is that we conducted this systematic review by rigorously following a published protocol with pre-specified analyses. Meta-regression including disease severity markers provides insight into the heterogeneous results of QoL found in earlier studies.

Our study comes with a number of limitations. First, not all studies reported values of all disease severity markers, which limited the number of studies included in our meta-regression analysis. Furthermore, not all potential modifiers of QoL in ADPKD could be included in this study. Earlier studies have shown that comorbidity, the use of pain medication, presence of a cerebral aneurysm, and lower education levels are associated with lower QoL in this population [16, 38]. Two qualitative studies showed that genetic guilt also might be an important factor that influences QoL [42, 43]. The lack of detail in the articles under study precluded systematic analysis of these factors.

Secondly, part of heterogeneity in QoL might also be explained by study design. Most included studies were intervention studies. Patients involved in these studies do not resemble the general ADPKD patient population. QoL of patients participating in intervention studies was generally lower compared to patients from observational studies, likely due to the reality that most interventional studies have included patients with larger livers. However, the large variability in disease severity markers enabled us to thoroughly investigate the impact of these factors on QoL in individuals with ADPKD.

Third, we could include only QoL data collected with a generic patient-reported outcome, which is likely less sensitive to detect disease burden than disease-specific measures [44]. On the other hand, an unpublished global observational study of 3409 individuals with ADPKD showed that the PCS of the SF-12, a short version of the SF-36, could differentiate QoL between CKD 1 and CKD 3a, while the disease-specific ADPKD Impact Scale could differentiate CKD 1 from CKD stage 3b [45]. This suggests that the PCS distinguishes between individuals with ADPKD in a slightly earlier phase than the disease-specific ADPKD Impact Scale. The MCS of the SF-12 was unable to differentiate between CKD 1 and other disease stages, indicating that this component score is insensitive to change in this disease population.

This systematic review on QoL in ADPKD clearly identified knowledge gaps. Data on QoL in patients with ADPKD and more advanced stages of CKD were insufficient to be assessed. As liver volume appears to impact QoL, clinicians should check liver disease severity periodically and consider liver volume reducing therapies in severe hepatomegaly as a strategy to improve patients’ wellbeing. Indeed, research shows that reduction of liver volume with somatostatin analogues improves QoL in severe polycystic liver disease [46]. However, currently data is lacking on the effect of other therapies on QoL. In patients without severe polycystic liver disease, QoL was negatively impacted by renal function. Vasopressin V_2_ receptor antagonists slow renal function decline, but additional studies should investigate whether the possible positive effect on QoL is counteracted by side effects such as aquaresis.

## Conclusion

In conclusion, there is limited representative data available on the impact of disease severity markers on QoL in ADPKD. Existing data showed that QoL of non-dialysis ADPKD patients is impaired compared to the general population. Large liver volume was the most important factor that diminishes QoL.

## Additional files

Additional file 1: Table S1. Example of the search strategy (MEDLINE). **Table S2.** Risk of bias assessment QoL studies. (DOCX 18 kb)

Additional file 2: Figure S1. Meta-regression analysis of the mental component score of the SF-36 with the factors (A) (e)GFR (ml/min/1.73m^2^) (B) Kidney volume in mL, (C) liver volume in mL and (D) total liver and kidney volume in mL. (TIFF 67 kb)

Additional file 3: Figure S2. Meta-regression analysis of the mental component score of the SF-36 with the factors (A) eGFR (ml/min/1.73m^2^) and (B) Kidney volume in studies with mild to moderate liver involvement. (TIFF 54 kb)

## Abbreviations

(e)GFR: (estimated) glomerular filtration rate
95% CI: 95% confidence interval
ADPKD: Autosomal dominant polycystic kidney disease
ADPLD: Autosomal dominant polycystic liver disease
ASN: American society of nephrology
CKD: Chronic kidney disease
CKD-EPI: Chronic kidney disease – epidemiology collaboration equation
ERA-EDTA: European renal association – European transplant and dialysis association
IQR: Interquartile range
MCS: Mental component score
MDRD: Modification of diet in renal disease equation
PCS: Physical component score
PRISMA: Preferred reporting items for systematic reviews and meta-analyses
QoL: Quality of life
SD: Standard deviation
SE: Standard error
WCN: World congress of nephrology
