# Supplementary material for: The effect of disease severity markers on quality of life in autosomal dominant polycystic kidney disease: a systematic review, meta-analysis and meta-regression
Source: BMC Nephrol. 2017 May 25;18:169. doi: 10.1186/s12882-017-0578-6 (PMC5445294; doi:10.1186/s12882-017-0578-6)
Supplement: Supplementary file 1 — Example of the search strategy (MEDLINE). Table S2. Risk of bias assessment QoL studies. (DOCX 18 kb) [file 12882_2017_578_MOESM1_ESM.docx]

**Additional file 1**

**Table S1.** Example of the search strategy (MEDLINE)

| **Ovid MEDLINE(R) In-Process & Other Non-Indexed Citations and Ovid MEDLINE(R)**1946 to Present | | | |
| --- | --- | --- | --- |
| **#** | **Searches** | **Results** | **Search Type** |
| 1 | exp kidney diseases, cystic/ | 13464 | Advanced |
| 2 | (((cystic or polycystic) adj3 (renal or kidney)) or pkd or pckd or adpkd).mp. [mp=title, abstract, original title, name of substance word, subject heading word, keyword heading word, protocol supplementary concept word, rare disease supplementary concept word, unique identifier] | 16952 | Advanced |
| 3 | 1 or 2 | 17577 | Advanced |
| 4 | exp kidney diseases, cystic/ph, pp, ah or (2 and (pathophysiol* or physiopathol* or function* or volume* or dysfunction*).mp.) [mp=title, abstract, original title, name of substance word, subject heading word, keyword heading word, protocol supplementary concept word, rare disease supplementary concept word, unique identifier] | 4516 | Advanced |
| 5 | 3 and (exp kidney function tests/ or exp kidney/ah, pp, ph) | 1131 | Advanced |
| 6 | 4 or 5 | 4830 | Advanced |
| 7 | (sf36 or "sf 36" or "short form 36").mp. [mp=title, abstract, original title, name of substance word, subject heading word, keyword heading word, protocol supplementary concept word, rare disease supplementary concept word, unique identifier] | 18256 | Advanced |
| 8 | quality of life/ or hrqol.mp. or "functional capacity".mp. or adl.mp. or activities of daily living/ [mp=title, abstract, original title, name of substance word, subject heading word, keyword heading word, protocol supplementary concept word, rare disease supplementary concept word, unique identifier] | 184841 | Advanced |
| 9 | (anxiety or depression or life expectancy or life style or social support or lifestyle).mp. or stress, psychological/ or distress*.mp. or fear*.mp. or family*.mp. or pain*.mp. or psychosocial*.mp. or discomfort*.mp. or symptom*.mp. or satiety.mp. or disability.mp. [mp=title, abstract, original title, name of substance word, subject heading word, keyword heading word, protocol supplementary concept word, rare disease supplementary concept word, unique identifier] | 2730834 | Advanced |
| 10 | (guilt or fatigue or sleep* or social life* or productiv* or intimacy or intimate).mp. or exp sexual dysfunctions, psychological/ or exp sexual dysfunction, physiological/ [mp=title, abstract, original title, name of substance word, subject heading word, keyword heading word, protocol supplementary concept word, rare disease supplementary concept word, unique identifier] | 339034 | Advanced |
| 11 | 6 and 7 | 7 | Advanced |
| 12 | or/8-10 | 3047299 | Advanced |
| 13 | 6 and (12 or px.fs.) | 924 | Advanced |
| 14 | limit 11 to (clinical trial, all or clinical trial, phase i or clinical trial, phase ii or clinical trial, phase iii or clinical trial, phase iv or clinical trial or comparative study or controlled clinical trial or evaluation studies or meta analysis or multicenter study or observational study or pragmatic clinical trial or randomized controlled trial) | 5 | Advanced |
| 15 | 13 and ("cross section*".mp. or follow-up studies/ or "health status".mp. or longitudinal*.mp.) [mp=title, abstract, original title, name of substance word, subject heading word, keyword heading word, protocol supplementary concept word, rare disease supplementary concept word, unique identifier] | 78 | Advanced |
| 16 | 13 and questionnaire*.mp. [mp=title, abstract, original title, name of substance word, subject heading word, keyword heading word, protocol supplementary concept word, rare disease supplementary concept word, unique identifier] | 23 | Advanced |
| 17 | 13 and (cohort* or prospective* or retrospective*).mp. [mp=title, abstract, original title, name of substance word, subject heading word, keyword heading word, protocol supplementary concept word, rare disease supplementary concept word, unique identifier] | 132 | Advanced |
| 18 | or/14-17 | 187 | Advanced |
| 19 | remove duplicates from 18 | 177 | Advanced |
| 20 | limit 19 to "all adult (19 plus years)" | 119 | Advanced |
| 21 | 19 and (adult* or "middle age*" or "young adult*" or elder* or older).mp. [mp=title, abstract, original title, name of substance word, subject heading word, keyword heading word, protocol supplementary concept word, rare disease supplementary concept word, unique identifier] | 129 | Advanced |
| 22 | 20 or 21 | 129 |  |

**Table S2.** Risk of bias assessment QoL studies

|  | Study level | | Outcome level | | | Conclusion |
| --- | --- | --- | --- | --- | --- | --- |
|  | **Study population selection** | **Completeness of reported results** | **Used PRO instrument** | **Recall period^a^** | **Response rate^b^** |  |
| Barros et al, 2011 | ─ | ─ | ─ | ─ | ─ | Low |
| Hogan et al, 2010 | ─ | ─ | ─ | ─ | ─ | Low |
| Keimpema et al, 2009 | ─ | ─ | ─ | ─ | ─ | Low |
| Lee et al, 2003 | + | ─ | ─ | ─ | + | High |
| Miskulin et al, 2014 | ─ | ─ | ─ | ─ | ─ | Low |
| Rizk et al, 2009 | ─ | ─ | ─ | ─ | ─ | Low |
| Simms et at, 2015 | ─ | ─ | ─ | ─ | ─ | Low |
| Suwabe et al, 2013 | ─ | ─ | ─ | ─ | ─ | Low |
| Temmerman et al, 2014 | ─ | ─ | ─ | ─ | ─ | Low |

Risk of bias summary of the included studies as judged by the authors. Definition of symbols: − low risk of bias; ? unclear risk of bias; + high risk of bias. ^a^Recall period longer than 1 month was defined as high risk of bias. [[41](#_ENREF_41)] ^b^Response rate ˂80% or 60-80% and no non responders analysis was defined as high risk of bias. [[42](#_ENREF_42)]
