# Supplementary figures and images for: The effect of disease severity markers on quality of life in autosomal dominant polycystic kidney disease: a systematic review, meta-analysis and meta-regression
Source: BMC Nephrol. 2017 May 25;18:169. doi: 10.1186/s12882-017-0578-6 (PMC5445294; doi:10.1186/s12882-017-0578-6)

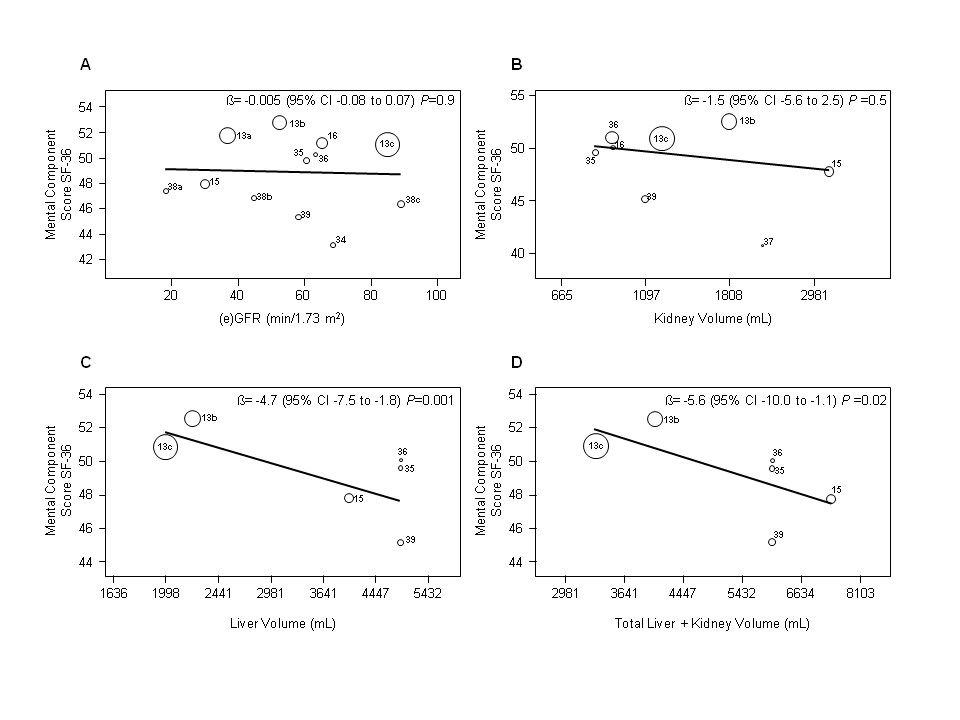

Supplement: Supplementary file 2 — Meta-regression analysis of the mental component score of the SF-36 with the factors (A) (e)GFR (ml/min/1.73m2) (B) Kidney volume in mL, (C) liver volume in mL and (D) total liver and kidney volume in mL. (TIFF 67 kb) [file 12882_2017_578_MOESM2_ESM.tif]

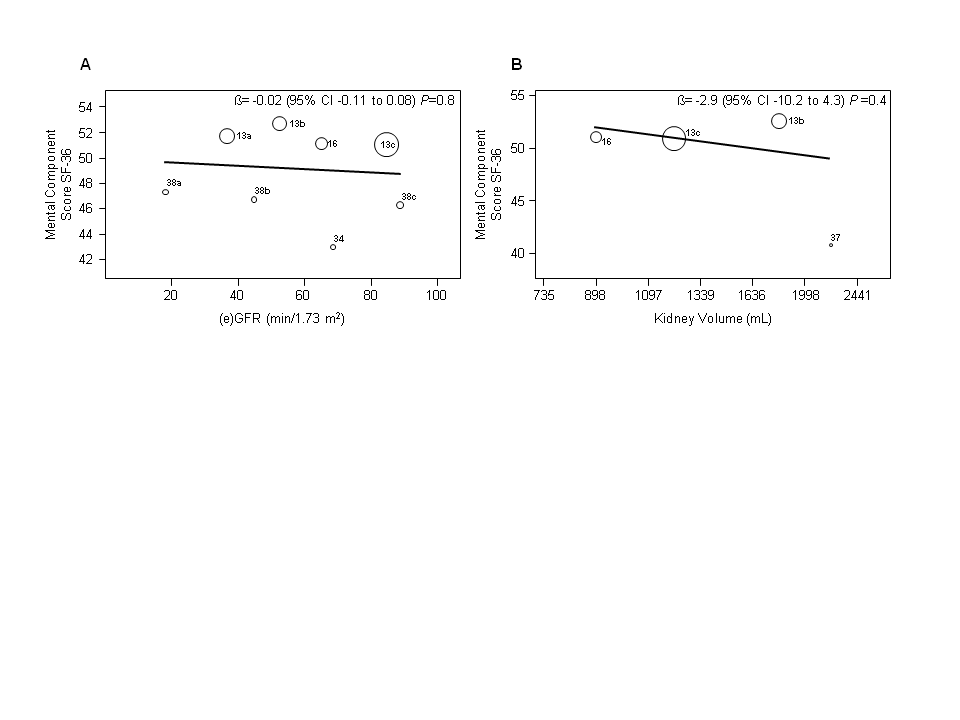

Supplement: Supplementary file 3 — Meta-regression analysis of the mental component score of the SF-36 with the factors (A) eGFR (ml/min/1.73m2) and (B) Kidney volume in studies with mild to moderate liver involvement. (TIFF 54 kb) [file 12882_2017_578_MOESM3_ESM.tif]
